# (2aR*,5S*,6aS*,8aS*,*E*)-Ethyl 5-hy­droxy-7,7,8a-trimethyl-8-oxo-2,2a,6,6a,7,8,8a,8b-octa­hydro-1*H*-penta­leno[1,6-*bc*]oxepine-4-carboxyl­ate

**DOI:** 10.1107/S1600536812046776

**Published:** 2012-11-24

**Authors:** Goverdhan Mehta, C. S. Ananda Kumar, Saikat Sen

**Affiliations:** a Department of Organic Chemistry, Indian Institute of Science, Bangalore 560 012, India

## Abstract

The title compound, C_17_H_24_O_5_, featuring a 2-carbeth­oxy-3-oxepanone unit in its intra­molecularly O—H⋯O hydrogen-bonded enol form, was obtained *via* [(CF_3_CO_2_)_2_Rh]_2_-catal­ysed intra­molecular O—H bond insertion in the α-diazo-ω-hy­droxy-β-ketoester, ethyl 4-[(1*S*,3a*S*,6*R*,6a*S*)-6-hy­droxy-2,2,3a-trimethyl-3-oxo-octa­hydro­penta­len-1-yl]-2-diazo-3-oxobutano­ate. The seven-membered oxacyclic ring, thus constructed on a *cis*-fused diquinane platform, was found to adopt a distorted boat–sofa conformation.

## Related literature

For rhodium carbenoid-mediated inter­molecular O—H inser­tion reactions and their application to natural product synthesis, see: Paulissen *et al.* (1973 ▶); Cox *et al.* (1994 ▶); Haigh (1994 ▶); Aller *et al.* (1995 ▶); Shi *et al.* (1995 ▶); Bulugahapitiya *et al.* (1997 ▶); Moody & Miller (1998 ▶); Nelson *et al.* (2000 ▶); Medeiros & Wood (2010 ▶); Freeman *et al.* (2010 ▶); Morton *et al.* (2012 ▶). For rhodium-catalysed intra­molecular O—H insertion reactions, see: Paulissen *et al.* (1974 ▶); Moyer *et al.* (1985 ▶); Moody & Taylor (1987 ▶); Heslin & Moody (1988 ▶); Davies *et al.* (1990 ▶); Moody *et al.* (1992 ▶); Sarabia-Garciá *et al.* (1994) ▶; Pad­wa & Sá (1999 ▶); Im *et al.* (2005 ▶). For reviews on rhodium-mediated C—H insertion reactions, see: Doyle *et al.* (2010 ▶); Davies & Morton (2011 ▶). For the con­struction of an angularly fused triquinane skeleton *via* Rh^II^-catalysed intra­molecular C—H insertion, see: Srikrishna *et al.* (2012 ▶). For the isolation and synthesis of penifulvin A, see: Shim *et al.* (2006 ▶); Gaich & Mulzer (2009 ▶); Mehta & Khan (2012 ▶). For the application of *p*-acetamido­benzene­sulfonyl azide as a diazo trans­fer reagent, see: Baum *et al.* (1987 ▶). For ring conformations, see: Cremer & Pople (1975 ▶); Boessenkool & Boeyens (1980 ▶).
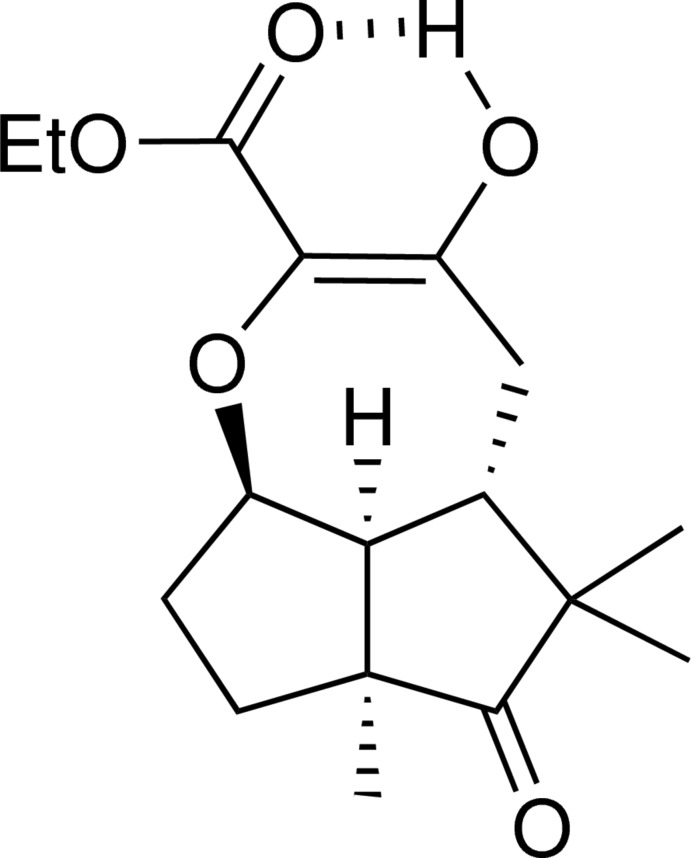


## Experimental

### 

#### Crystal data

C_17_H_24_O_5_
*M* *_r_* = 308.36
Orthorhombic, 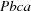

*a* = 8.447 (5) Å
*b* = 18.454 (14) Å
*c* = 21.735 (15) Å
*V* = 3388 (4) Å^3^
*Z* = 8
Mo *K*α radiation
μ = 0.09 mm^−1^
*T* = 291 K
0.20 × 0.18 × 0.08 mm

#### Data collection

Bruker APEXII CCD diffractometer
Absorption correction: multi-scan (*SADABS*; Sheldrick, 2003 ▶) *T*_min_ = 0.983, *T*_max_ = 0.993
14606 measured reflections
3153 independent reflections
1408 reflections with *I* > 2σ(*I*)
*R*_int_ = 0.087

#### Refinement

*R*[*F* ^2^ > 2σ(*F* ^2^)] = 0.053
*wR*(*F* ^2^) = 0.149
*S* = 0.89
3153 reflections
207 parameters
H atoms treated by a mixture of independent and constrained refinement
Δρ_max_ = 0.17 e Å^−3^
Δρ_min_ = −0.17 e Å^−3^

### 

Data collection: *APEX2* (Bruker, 2007 ▶); cell refinement: *SAINT* (Bruker, 2007 ▶); data reduction: *SAINT*; program(s) used to solve structure: *SIR92* (Altomare *et al.*, 1994 ▶); program(s) used to refine structure: *SHELXL97* (Sheldrick, 2008 ▶); molecular graphics: *ORTEP-3 for Windows* (Farrugia, 2012 ▶); software used to prepare material for publication: *PLATON* (Spek, 2009 ▶).

## Supplementary Material

Crystal structure: contains datablock(s) global, I. DOI: 10.1107/S1600536812046776/ds2220sup1.cif

Structure factors: contains datablock(s) I. DOI: 10.1107/S1600536812046776/ds2220Isup2.hkl

Additional supplementary materials:  crystallographic information; 3D view; checkCIF report

## Figures and Tables

**Table 1:** Hydrogen-bond geometry (Å, °)

*D*—H⋯*A*	*D*—H	H⋯*A*	*D*⋯*A*	*D*—H⋯*A*
O2—H2*O*⋯O4	0.93 (3)	1.69 (3)	2.565 (4)	155 (3)

**Fig. 1.**
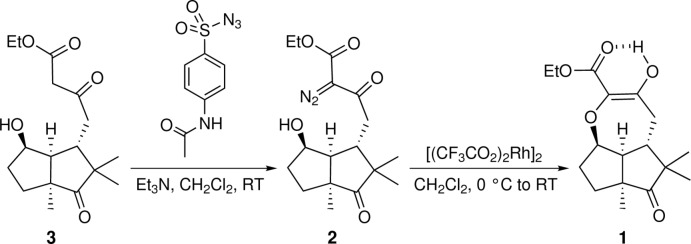
Preparation of the title compound 1 from the β-ketoester 3.

**Fig. 2.**
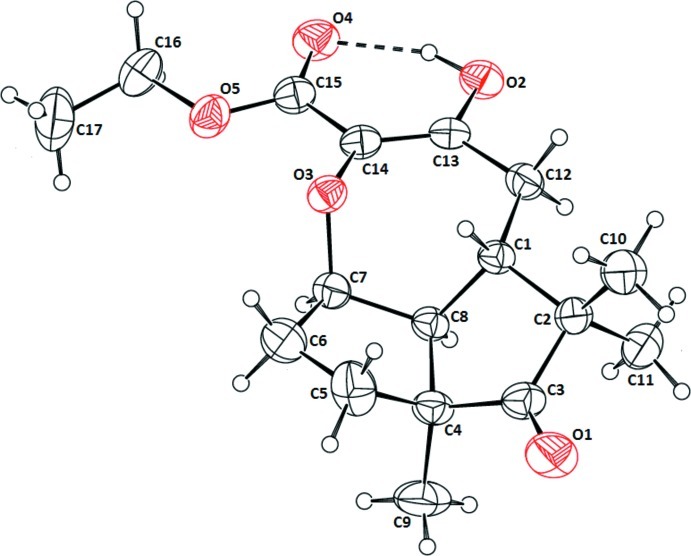
View of the title compound 1, with the atom numbering scheme of the asymmetric unit. Displacement ellipsoids for non-H atoms are drawn at the 30% probability level.

## supplementary crystallographic information

### Comment

Rhodium carbenoid mediated O—H insertion provides a facile means of transforming diazo-compounds into diverse range of functionalized ethers (Paulissen, *et al.*, 1973; Cox, *et al.* 1994; Haigh, 1994; Aller *et al.*, 1995; Shi *et al.*, 1995; Bulugahapitiya *et al.*, 1997; Moody & Miller, 1998; Morton *et al.*, 2012). Hence the methodology has proven to be a useful stratagem in the synthetic acquisition of several natural products (Nelson *et al.*, 2000; Medeiros & Wood, 2010; Freeman, *et al.* 2010). While not as extensively utilized or studied as the intermolecular variants, intramolecular interception of rhodium carbenoids by hydroxy nucleophiles can, nevertheless, afford an effective route to cyclic ethers and lactones (Paulissen *et al.*, 1974; Moyer *et al.*, 1985; Moody & Taylor, 1987; Heslin & Moody, 1988; Moody *et al.* 1992; Sarabia-García *et al.*, 1994; Padwa & Sá, 1999; Im *et al.*, 2005). Indeed, studies by Moody and co-workers have shown that rhodium(II) acetate catalysed cyclization in diazoalcohols may even be employed as a practical method for accessing medium-ring oxacycles - oxepanes, in particular, wherein interference from competing C—H insertion reactions do not appear to be significant (Heslin & Moody, 1988; Davies *et al.*, 1990).

Against this background, we report herein the crystal structure of the title compound 1, a 2-carbethoxy-3-oxepanone embedded in a tricyclic framework, that was obtained as the sole isolable product in the rhodium(II) trifluroacetate mediated decomposition of the α-diazo-ω-hydroxy-β-ketoester 2 (Figure 1). Originally envisaged as an entry point to an angularly fused triquinane skeleton *via* Rh(II) catalyzed intramolecular C—H insertion (Doyle *et al.*, 2010; Davies & Morton, 2011; Srikrishna *et al.*, 2012) *en route* to the natural product penifulvin A (Shim *et al.*, 2006; Gaich & Mulzer, 2009; Mehta & Khan, 2012), the diazoester 2 was prepared from the β-ketoester 3*via* a diazo transfer reaction to the activated methylene group in 3 (Baum *et al.*, 1987).

The crystal structure of 1 was solved and refined in the centrosymmetric orthorhombic space group *Pbcn* (*Z* = 8). The 2-carbethoxy-3-oxepanone moiety in 1 was found to exist in the intramolecularly O—H···O hydrogen bonded enol form (Figure 2). As indicated by its puckering parameters (q_2_ = 0.915 (3) Å, q_3_ = 0.310 (3) Å, φ_2_ = 193.59 (17)°, φ_3_ = 118.9 (5)°, Q_T_ = 0.967 (2) Å), the seven-membered oxacyclic ring adopted a distorted boat-sofa conformation (Cremer & Pople, 1975; Boessenkool & Boeyens, 1980). Crystal packing in 1 was effected primarily *via* the agency of weak van der Waals interactions, though short C—H···O contacts (C8—H8···O2) could be discerned among the molecules.

### Experimental

As shown in Figure 1, the title compound 1 was prepared from the the β-ketoester 3*via* the intermediate diazoester 2. Thus, 3, upon treatment with *p*-acetamidobenzenesulfonyl azide and triethylamine, afforded 2. The α-diazo-ω-hydroxy-β-ketoester 2 underwent smooth cyclization in presence of catalytic rhodium(II) trifluoroacetate dimer to deliver the oxepanone 1, which crystallized as thin colorless plates from 1:1 dichloromethane-hexanes.

### Refinement

The methine (CH) and methylene (CH_2_) H atoms were placed in geometrically idealized positions and allowed to ride on their parent atoms with C—H distances 0.93 and 0.97 Å respectively, and *U*_iso_(H) = 1.2*U*_eq_(C). The methyl (CH_3_) hydrogen atoms were constrained to an ideal geometry with C—H distances as 0.96 Å and *U*_iso_(H) = 1.5*U*_eq_(C). During refinement, each methyl group was however allowed to rotate freely about its C—C bond. The position of the hydroxyl hydrogen atom was refined freely, along with an isotropic displacement parameter.

### Crystal data

**Table:**

C_17_H_24_O_5_	*F*(000) = 1328
*M**_r_* = 308.36	*D*_x_ = 1.209 Mg m^−^^3^
Orthorhombic, *P**b**c**a*	Mo *K*α radiation, λ = 0.71073 Å
Hall symbol: -P 2ac 2ab	Cell parameters from 939 reflections
*a* = 8.447 (5) Å	θ = 2.8–19.5°
*b* = 18.454 (14) Å	µ = 0.09 mm^−^^1^
*c* = 21.735 (15) Å	*T* = 291 K
*V* = 3388 (4) Å^3^	Plate, colorless
*Z* = 8	0.20 × 0.18 × 0.08 mm

### Data collection

**Table:**

Bruker APEXII CCD diffractometer	3153 independent reflections
Radiation source: fine-focus sealed tube	1408 reflections with *I* > 2σ(*I*)
Graphite monochromator	*R*_int_ = 0.087
φ and ω scans	θ_max_ = 25.5°, θ_min_ = 1.9°
Absorption correction: multi-scan (*SADABS*; Sheldrick, 2003)	*h* = −10→7
*T*_min_ = 0.983, *T*_max_ = 0.993	*k* = −22→18
14606 measured reflections	*l* = −26→26

### Refinement

**Table:**

Refinement on *F*^2^	Primary atom site location: structure-invariant direct methods
Least-squares matrix: full	Secondary atom site location: difference Fourier map
*R*[*F*^2^ > 2σ(*F*^2^)] = 0.053	Hydrogen site location: inferred from neighbouring sites
*wR*(*F*^2^) = 0.149	H atoms treated by a mixture of independent and constrained refinement
*S* = 0.89	*w* = 1/[σ^2^(*F*_o_^2^) + (0.0717*P*)^2^] where *P* = (*F*_o_^2^ + 2*F*_c_^2^)/3
3153 reflections	(Δ/σ)_max_ < 0.001
207 parameters	Δρ_max_ = 0.17 e Å^−^^3^
0 restraints	Δρ_min_ = −0.17 e Å^−^^3^

### Special details

**Table:**

Geometry. All s.u.'s (except the s.u. in the dihedral angle between two l.s. planes) are estimated using the full covariance matrix. The cell s.u.'s are taken into account individually in the estimation of s.u.'s in distances, angles and torsion angles; correlations between s.u.'s in cell parameters are only used when they are defined by crystal symmetry. An approximate (isotropic) treatment of cell s.u.'s is used for estimating s.u.'s involving l.s. planes.
Refinement. Refinement of *F*^2^ against ALL reflections. The weighted *R*-factor *wR* and goodness of fit *S* are based on *F*^2^, conventional *R*-factors *R* are based on *F*, with *F* set to zero for negative *F*^2^. The threshold expression of *F*^2^ > 2σ(*F*^2^) is used only for calculating *R*-factors(gt) *etc*. and is not relevant to the choice of reflections for refinement. *R*-factors based on *F*^2^ are statistically about twice as large as those based on *F*, and *R*- factors based on ALL data will be even larger.

### Fractional atomic coordinates and isotropic or equivalent isotropic displacement parameters (Å^2^)

**Table:**

	*x*	*y*	*z*	*U*_iso_*/*U*_eq_	
O1	0.1027 (3)	0.15812 (15)	1.02261 (10)	0.0990 (9)	
O2	0.0947 (2)	0.13477 (12)	0.69261 (9)	0.0655 (6)	
H2O	0.125 (4)	0.0976 (18)	0.6660 (15)	0.090 (12)*	
O3	0.1391 (2)	−0.00529 (10)	0.80956 (7)	0.0545 (5)	
O4	0.1993 (2)	0.01550 (12)	0.64764 (8)	0.0717 (6)	
O5	0.2612 (2)	−0.07327 (12)	0.71407 (8)	0.0678 (6)	
C1	0.0661 (3)	0.12205 (14)	0.86389 (11)	0.0471 (7)	
H1	−0.0058	0.0810	0.8699	0.057*	
C2	0.0332 (3)	0.17697 (15)	0.91600 (12)	0.0544 (8)	
C3	0.1256 (3)	0.14428 (17)	0.96932 (13)	0.0623 (8)	
C4	0.2513 (3)	0.09177 (16)	0.94596 (12)	0.0568 (8)	
C5	0.2035 (5)	0.01234 (18)	0.95976 (14)	0.0888 (11)	
H5A	0.2518	−0.0039	0.9978	0.107*	
H5B	0.0895	0.0083	0.9637	0.107*	
C6	0.2602 (5)	−0.03189 (19)	0.90749 (14)	0.0906 (12)	
H6A	0.1898	−0.0726	0.9006	0.109*	
H6B	0.3655	−0.0504	0.9158	0.109*	
C7	0.2627 (3)	0.01697 (15)	0.85202 (12)	0.0575 (8)	
H7	0.3661	0.0139	0.8317	0.069*	
C8	0.2347 (3)	0.09461 (14)	0.87520 (11)	0.0474 (7)	
H8	0.3125	0.1280	0.8574	0.057*	
C9	0.4153 (4)	0.1103 (2)	0.97087 (15)	0.0970 (12)	
H9A	0.4449	0.1580	0.9573	0.145*	
H9B	0.4908	0.0757	0.9559	0.145*	
H9C	0.4133	0.1090	1.0150	0.145*	
C10	−0.1415 (4)	0.1859 (2)	0.93120 (14)	0.0795 (11)	
H10A	−0.1868	0.1394	0.9400	0.119*	
H10B	−0.1952	0.2072	0.8967	0.119*	
H10C	−0.1527	0.2168	0.9664	0.119*	
C11	0.1093 (4)	0.25222 (16)	0.90386 (15)	0.0781 (10)	
H11A	0.1031	0.2811	0.9405	0.117*	
H11B	0.0537	0.2761	0.8711	0.117*	
H11C	0.2183	0.2460	0.8925	0.117*	
C12	0.0415 (3)	0.14979 (16)	0.79823 (11)	0.0569 (8)	
H12A	−0.0704	0.1595	0.7925	0.068*	
H12B	0.0971	0.1955	0.7940	0.068*	
C13	0.0951 (3)	0.10042 (16)	0.74780 (12)	0.0510 (7)	
C14	0.1445 (3)	0.03157 (16)	0.75362 (11)	0.0512 (7)	
C15	0.2038 (3)	−0.00865 (17)	0.70042 (13)	0.0572 (8)	
C16	0.3258 (4)	−0.11534 (19)	0.66252 (15)	0.0833 (11)	
H16A	0.2406	−0.1329	0.6366	0.100*	
H16B	0.3950	−0.0852	0.6378	0.100*	
C17	0.4146 (6)	−0.1765 (2)	0.68814 (18)	0.1264 (17)	
H17A	0.4935	−0.1587	0.7161	0.190*	
H17B	0.4650	−0.2027	0.6554	0.190*	
H17C	0.3435	−0.2081	0.7097	0.190*	

### Atomic displacement parameters (Å^2^)

**Table:**

	*U*^11^	*U*^22^	*U*^33^	*U*^12^	*U*^13^	*U*^23^
O1	0.113 (2)	0.137 (2)	0.0465 (13)	0.0369 (16)	−0.0008 (12)	−0.0152 (14)
O2	0.0791 (15)	0.0722 (16)	0.0452 (12)	0.0013 (12)	−0.0053 (10)	0.0083 (12)
O3	0.0605 (13)	0.0555 (13)	0.0474 (11)	−0.0043 (9)	0.0016 (9)	−0.0012 (9)
O4	0.0820 (16)	0.0883 (16)	0.0448 (12)	0.0032 (12)	0.0027 (10)	−0.0031 (11)
O5	0.0850 (16)	0.0645 (14)	0.0540 (12)	0.0051 (12)	0.0061 (10)	−0.0092 (11)
C1	0.0407 (18)	0.0550 (17)	0.0456 (16)	−0.0004 (13)	−0.0013 (12)	−0.0012 (13)
C2	0.0495 (19)	0.060 (2)	0.0541 (17)	0.0038 (15)	−0.0001 (13)	−0.0068 (15)
C3	0.063 (2)	0.078 (2)	0.0463 (18)	−0.0012 (17)	−0.0008 (15)	−0.0052 (16)
C4	0.054 (2)	0.073 (2)	0.0438 (15)	0.0052 (16)	−0.0048 (13)	−0.0029 (15)
C5	0.117 (3)	0.085 (3)	0.064 (2)	0.019 (2)	0.0199 (19)	0.023 (2)
C6	0.128 (3)	0.076 (2)	0.067 (2)	0.010 (2)	−0.022 (2)	0.013 (2)
C7	0.058 (2)	0.0618 (19)	0.0524 (16)	0.0087 (15)	−0.0061 (14)	−0.0047 (15)
C8	0.0385 (17)	0.0557 (18)	0.0481 (15)	0.0009 (14)	−0.0004 (12)	−0.0002 (13)
C9	0.060 (2)	0.151 (4)	0.080 (2)	0.010 (2)	−0.0213 (18)	−0.031 (2)
C10	0.059 (2)	0.103 (3)	0.076 (2)	0.0165 (19)	0.0071 (17)	−0.0200 (19)
C11	0.091 (2)	0.063 (2)	0.081 (2)	−0.0007 (19)	−0.0015 (18)	−0.0131 (18)
C12	0.059 (2)	0.0625 (19)	0.0496 (16)	0.0101 (15)	−0.0062 (13)	0.0010 (14)
C13	0.0500 (18)	0.063 (2)	0.0404 (15)	−0.0043 (15)	−0.0073 (12)	0.0002 (15)
C14	0.0520 (19)	0.063 (2)	0.0388 (15)	−0.0052 (15)	0.0005 (12)	−0.0016 (14)
C15	0.054 (2)	0.064 (2)	0.0535 (19)	−0.0055 (17)	0.0008 (14)	−0.0021 (16)
C16	0.100 (3)	0.077 (2)	0.073 (2)	0.005 (2)	0.0090 (19)	−0.026 (2)
C17	0.194 (5)	0.076 (3)	0.109 (3)	0.047 (3)	0.008 (3)	−0.010 (2)

### Geometric parameters (Å, º)

**Table:**

O1—C3	1.202 (3)	C6—H6B	0.9700
O2—C13	1.357 (3)	C7—C8	1.537 (4)
O2—H2O	0.93 (3)	C7—H7	0.9800
O3—C14	1.394 (3)	C8—H8	0.9800
O3—C7	1.452 (3)	C9—H9A	0.9600
O4—C15	1.231 (3)	C9—H9B	0.9600
O5—C15	1.321 (3)	C9—H9C	0.9600
O5—C16	1.468 (3)	C10—H10A	0.9600
C1—C12	1.530 (3)	C10—H10B	0.9600
C1—C8	1.531 (3)	C10—H10C	0.9600
C1—C2	1.545 (3)	C11—H11A	0.9600
C1—H1	0.9800	C11—H11B	0.9600
C2—C10	1.521 (4)	C11—H11C	0.9600
C2—C3	1.522 (4)	C12—C13	1.496 (4)
C2—C11	1.553 (4)	C12—H12A	0.9700
C3—C4	1.525 (4)	C12—H12B	0.9700
C4—C9	1.526 (4)	C13—C14	1.343 (4)
C4—C8	1.545 (4)	C14—C15	1.462 (4)
C4—C5	1.550 (4)	C16—C17	1.465 (5)
C5—C6	1.479 (4)	C16—H16A	0.9700
C5—H5A	0.9700	C16—H16B	0.9700
C5—H5B	0.9700	C17—H17A	0.9600
C6—C7	1.505 (4)	C17—H17B	0.9600
C6—H6A	0.9700	C17—H17C	0.9600
			
C13—O2—H2O	102 (2)	C4—C8—H8	110.7
C14—O3—C7	113.1 (2)	C4—C9—H9A	109.5
C15—O5—C16	116.3 (2)	C4—C9—H9B	109.5
C12—C1—C8	112.7 (2)	H9A—C9—H9B	109.5
C12—C1—C2	116.1 (2)	C4—C9—H9C	109.5
C8—C1—C2	105.5 (2)	H9A—C9—H9C	109.5
C12—C1—H1	107.4	H9B—C9—H9C	109.5
C8—C1—H1	107.4	C2—C10—H10A	109.5
C2—C1—H1	107.4	C2—C10—H10B	109.5
C10—C2—C3	112.0 (2)	H10A—C10—H10B	109.5
C10—C2—C1	113.9 (2)	C2—C10—H10C	109.5
C3—C2—C1	101.9 (2)	H10A—C10—H10C	109.5
C10—C2—C11	110.0 (3)	H10B—C10—H10C	109.5
C3—C2—C11	105.7 (2)	C2—C11—H11A	109.5
C1—C2—C11	112.8 (2)	C2—C11—H11B	109.5
O1—C3—C2	124.6 (3)	H11A—C11—H11B	109.5
O1—C3—C4	124.6 (3)	C2—C11—H11C	109.5
C2—C3—C4	110.8 (2)	H11A—C11—H11C	109.5
C3—C4—C9	111.8 (2)	H11B—C11—H11C	109.5
C3—C4—C8	104.3 (2)	C13—C12—C1	116.0 (2)
C9—C4—C8	115.3 (2)	C13—C12—H12A	108.3
C3—C4—C5	110.8 (2)	C1—C12—H12A	108.3
C9—C4—C5	112.3 (3)	C13—C12—H12B	108.3
C8—C4—C5	101.6 (2)	C1—C12—H12B	108.3
C6—C5—C4	106.8 (3)	H12A—C12—H12B	107.4
C6—C5—H5A	110.4	C14—C13—O2	121.7 (3)
C4—C5—H5A	110.4	C14—C13—C12	127.0 (3)
C6—C5—H5B	110.4	O2—C13—C12	111.3 (3)
C4—C5—H5B	110.4	C13—C14—O3	122.2 (2)
H5A—C5—H5B	108.6	C13—C14—C15	120.8 (3)
C5—C6—C7	106.8 (3)	O3—C14—C15	117.0 (3)
C5—C6—H6A	110.4	O4—C15—O5	123.2 (3)
C7—C6—H6A	110.4	O4—C15—C14	122.9 (3)
C5—C6—H6B	110.4	O5—C15—C14	114.0 (3)
C7—C6—H6B	110.4	C17—C16—O5	107.9 (3)
H6A—C6—H6B	108.6	C17—C16—H16A	110.1
O3—C7—C6	109.2 (3)	O5—C16—H16A	110.1
O3—C7—C8	111.2 (2)	C17—C16—H16B	110.1
C6—C7—C8	107.1 (2)	O5—C16—H16B	110.1
O3—C7—H7	109.8	H16A—C16—H16B	108.4
C6—C7—H7	109.8	C16—C17—H17A	109.5
C8—C7—H7	109.8	C16—C17—H17B	109.5
C1—C8—C7	113.5 (2)	H17A—C17—H17B	109.5
C1—C8—C4	104.8 (2)	C16—C17—H17C	109.5
C7—C8—C4	106.3 (2)	H17A—C17—H17C	109.5
C1—C8—H8	110.7	H17B—C17—H17C	109.5
C7—C8—H8	110.7		
			
C12—C1—C2—C10	79.9 (3)	C2—C1—C8—C4	35.4 (3)
C8—C1—C2—C10	−154.5 (2)	O3—C7—C8—C1	16.3 (3)
C12—C1—C2—C3	−159.2 (2)	C6—C7—C8—C1	−103.0 (3)
C8—C1—C2—C3	−33.6 (3)	O3—C7—C8—C4	131.0 (2)
C12—C1—C2—C11	−46.3 (3)	C6—C7—C8—C4	11.7 (3)
C8—C1—C2—C11	79.3 (3)	C3—C4—C8—C1	−22.2 (3)
C10—C2—C3—O1	−38.1 (4)	C9—C4—C8—C1	−145.2 (3)
C1—C2—C3—O1	−160.2 (3)	C5—C4—C8—C1	93.0 (3)
C11—C2—C3—O1	81.8 (4)	C3—C4—C8—C7	−142.7 (2)
C10—C2—C3—C4	142.1 (3)	C9—C4—C8—C7	94.4 (3)
C1—C2—C3—C4	20.0 (3)	C5—C4—C8—C7	−27.4 (3)
C11—C2—C3—C4	−98.1 (3)	C8—C1—C12—C13	49.8 (3)
O1—C3—C4—C9	−53.5 (4)	C2—C1—C12—C13	171.6 (2)
C2—C3—C4—C9	126.4 (3)	C1—C12—C13—C14	8.5 (4)
O1—C3—C4—C8	−178.7 (3)	C1—C12—C13—O2	−169.0 (2)
C2—C3—C4—C8	1.1 (3)	O2—C13—C14—O3	−177.2 (2)
O1—C3—C4—C5	72.7 (4)	C12—C13—C14—O3	5.5 (4)
C2—C3—C4—C5	−107.5 (3)	O2—C13—C14—C15	1.5 (4)
C3—C4—C5—C6	144.6 (3)	C12—C13—C14—C15	−175.8 (3)
C9—C4—C5—C6	−89.5 (3)	C7—O3—C14—C13	−75.6 (3)
C8—C4—C5—C6	34.3 (3)	C7—O3—C14—C15	105.7 (3)
C4—C5—C6—C7	−28.1 (4)	C16—O5—C15—O4	1.9 (4)
C14—O3—C7—C6	−172.9 (2)	C16—O5—C15—C14	−178.6 (2)
C14—O3—C7—C8	69.1 (3)	C13—C14—C15—O4	−6.2 (4)
C5—C6—C7—O3	−110.5 (3)	O3—C14—C15—O4	172.5 (2)
C5—C6—C7—C8	10.0 (4)	C13—C14—C15—O5	174.3 (3)
C12—C1—C8—C7	−81.4 (3)	O3—C14—C15—O5	−7.0 (4)
C2—C1—C8—C7	151.0 (2)	C15—O5—C16—C17	167.3 (3)
C12—C1—C8—C4	163.1 (2)		

### Hydrogen-bond geometry (Å, º)

**Table:**

*D*—H···*A*	*D*—H	H···*A*	*D*···*A*	*D*—H···*A*
O2—H2*O*···O4	0.93 (3)	1.69 (3)	2.565 (4)	155 (3)
